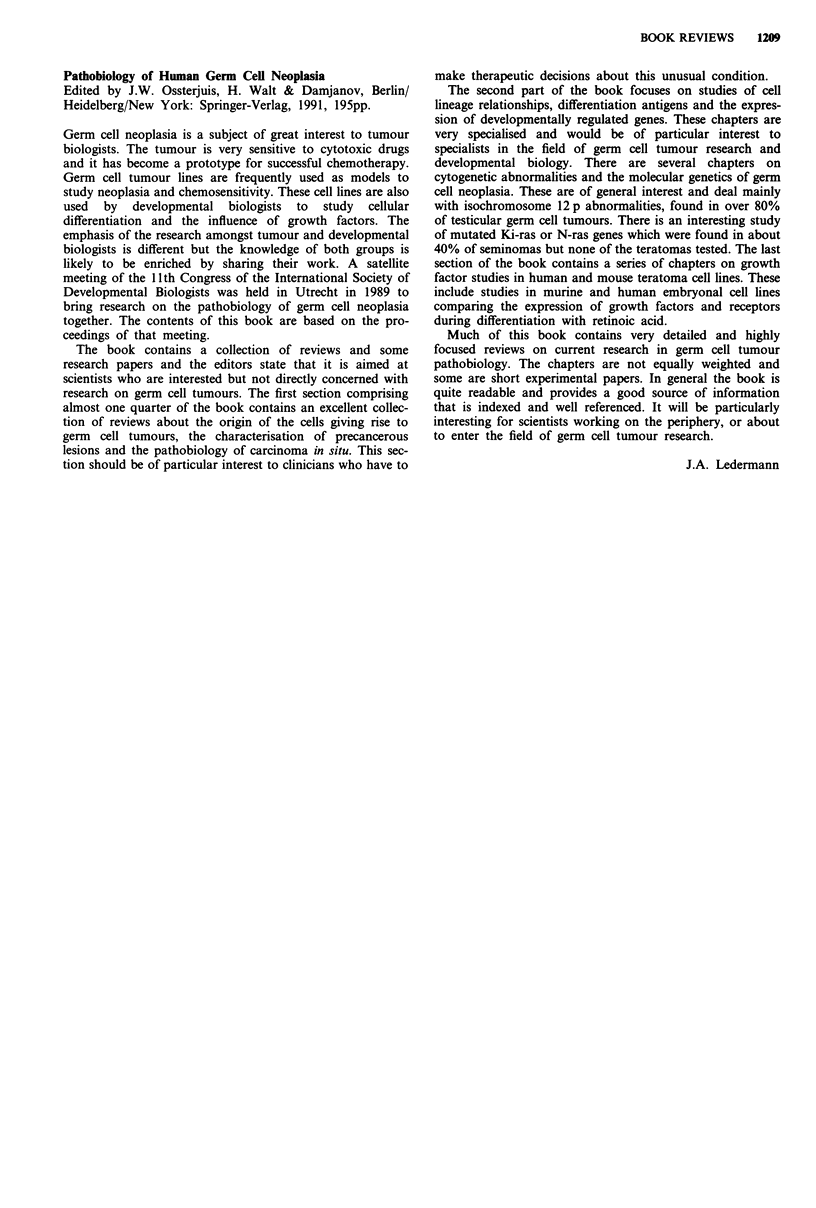# Pathobiology of Human Germ Cell Neoplasia

**Published:** 1992-12

**Authors:** J.A. Ledermann


**Pathobiology of Human Germ Cell Neoplasia**
Edited by J.W. Ossterjuis, H. Walt & Damjanov, Berlin/Heidelberg/New York: Springer-Verlag, 1991, 195pp.

Germ cell neoplasia is a subject of great interest to tumour biologists. The tumour is very sensitive to cytotoxic drugs and it has become a prototype for successful chemotherapy. Germ cell tumour lines are frequently used as models to study neoplasia and chemosensitivity. These cell lines are also used by developmental biologists to study cellular differentiation and the influence of growth factors. The emphasis of the research amongst tumour and developmental biologists is different but the knowledge of both groups is likely to be enriched by sharing their work. A satellite meeting of the 11th Congress of the International Society of Developmental Biologists was held in Utrecht in 1989 to bring research on the pathobiology of germ cell neoplasia together. The contents of this book are based on the proceedings of that meeting.

The book contains a collection of reviews and some research papers and the editors state that it is aimed at scientists who are interested but not directly concerned with research on germ cell tumours. The first section comprising almost one quarter of the book contains an excellent collection of reviews about the origin of the cells giving rise to germ cell tumours, the characterisation of precancerous lesions and the pathobiology of carcinoma *in situ*. This section should be of particular interest to clinicians who have to make therapeutic decisions about this unusual condition.

The second part of the book focuses on studies of cell lineage relationships, differentiation antigens and the expression of developmentally regulated genes. These chapters are very specialised and would be of particular interest to specialists in the field of germ cell tumour research and developmental biology. There are several chapters on cytogenetic abnormalities and the molecular genetics of germ cell neoplasia. These are of general interest and deal mainly with isochromosome 12 p abnormalities, found in over 80% of testicular germ cell tumours. There is an interesting study of mutated Ki-ras or N-ras genes which were found in about 40% of seminomas but none of the teratomas tested. The last section of the book contains a series of chapters on growth factor studies in human and mouse teratoma cell lines. These include studies in murine and human embryonal cell lines comparing the expression of growth factors and receptors during differentiation with retinoic acid.

Much of this book contains very detailed and highly focused reviews on current research in germ cell tumour pathobiology. The chapters are not equally weighted and some are short experimental papers. In general the book is quite readable and provides a good source of information that is indexed and well referenced. It will be particularly interesting for scientists working on the periphery, or about to enter the field of germ cell tumour research.

J.A. Ledermann